# Pyrenophora tritici-repentis population structure in the Republic of Kazakhstan and identification of wheat germplasm resistant to tan spot

**DOI:** 10.18699/VJ20.666

**Published:** 2020-11

**Authors:** A.M. Kokhmetova, N.M. Kovalenko, M.T. Kumarbaeva

**Affiliations:** Institute of Plant Biology and Biotechnology, Almaty, Kazakhstan Kazakh National Agrarian University, Almaty, Kazakhstan; All-Russian Research Institute of Plant Protection, Pushkin, St. Petersburg, Russia; Institute of Plant Biology and Biotechnology, Almaty, Kazakhstan Kazakh National Agrarian University, Almaty, Kazakhstan

**Keywords:** wheat, tan spot, races, molecular markers, Tsn1, ToxA, пшеница, пиренофороз, расы, молекулярные маркеры, Tsn1, ToxA

## Abstract

Pyrenophora tritici-repentis is a causative agent of tan spot in wheat. In recent years, there has been an increasing spread and harmfulness of wheat tan spot. The aim of the research was to study the racial composition of the P. tritici-repentis population in the Republic of Kazakhstan. A collection of 30 common wheat accessions, including promising lines and cultivars from Kazakhstan and CIMMYT–ICARDA, was assessed for resistance to P. tritici- repentis in a greenhouse and characterized using the Xfcp623 molecular marker, diagnostic for the Tsn1 gene. Monosporic isolates of P. tritici-repentis isolated from the southeastern region were assigned to certain races based on the manifestation of symptoms of necrosis/chlorosis on standard differentials (Glenlea, 6B662, 6B365). Five races of P. tritici-repentis have been identified, including races 1, 2, 3, 7 and 8. It has been shown that races 1 and 8 of P. tritici-repentis are dominant. As a result of the analysis of the frequency of occurrence of the P. tritici-repentis races, it was found that race 1 (50 %) producing Ptr ToxA and Ptr ToxB and race 8 (35 %) producing Ptr ToxA, Ptr ToxB and Ptr ToxC turned out to be dominant. From a practical point of view, of greatest interest are 16 wheat samples, which demonstrated resistance to race 1 and confirmed insensitivity to Ptr ToxA in a molecular screening. These include eight Kazakhstani (4_PSI, 10204_2_KSI, 10204_3_KSI, 10205_2_KSI, 10205_3_KSI, 605_SP2, 632_SP2, Dana) and seven foreign lines (KR11-20, KR11-03, KR11-9014, 11KR-13, KR11-9025, KR12-07, GN-68/2003). The results of this study are of interest in wheat breeding programs for tan spot resistance.

## Abstract (ru)

Возбудитель пиренофороза Pyrenophora tritici-repentis – одна из наиболее вредоносных бо- лезней листовых пятнистостей пшеницы. В последние годы отмечаются нарастающее распространение и вредоносность пиренофороза в Казахстане. Расовый состав P. tritici-repentis претерпевает изменения из-за климатических и средовых флуктуаций, а также из-за все более усиливающейся тенденции воз- делывания одних и тех же сортов пшеницы на больших территориях. В настоящее время имеется лишь ограниченная информация о расовой структуре популяции P. tritici-repentis в Казахстане. Целью исследо- ваний были изучение популяций P. tritici-repentis по расовому составу на юго-востоке Республики Казах- стан, а также идентификация устойчивых к пиренофорозу образцов пшеницы. Коллекция из 30 образцов мягкой пшеницы, включающая перспективные линии и сорта из Казахстана и из международных центров CIMMYT и ICARDA, была подвергнута оценке устойчивости к возбудителю пиренофороза в теплице и охарактеризована с использованием молекулярного маркера Xfcp623, диагностического для гена Tsn1. Моноконидиальные изоляты P. tritici-repentis, выделенные из популяции патогена юго-восточного региона, были отнесены к определенным расам на основе проявления симптомов некроза/хлороза с исполь- зованием стандартных образцов-дифференциаторов (Glenlea, 6В662, 6В365). Идентифицировано пять рас P. tritici-repentis, включающих расы 1, 2, 3, 7 и 8. Показано, что доминируют расы 1 и 8 P. tritici-repentis. В результате анализа частоты встречаемости рас возбудителя желтой пятнистости P. tritici-repentis уста- новлено, что доминирующей оказалась раса 1 (50 %), продуцирующая Ptr ToxA и Ptr ToxB, и раса 8 (35 %), продуцирующая Ptr ToxA, Ptr ToxB и Ptr ToxС. С практической точки зрения наибольший интерес представ- ляют 16 образцов пшеницы, которые демонстрировали устойчивость к расе 1 и подтвердили нечувстви- тельность к токсину Ptr ToxA при молекулярном скрининге. К ним относятся восемь казахстанских линий: 4_PSI, 10204_2_KSI, 10204_3_KSI, 10205_2_KSI, 10205_3_KSI, 605_SP2, 632_SP2, Dana и семь зарубежных линий: KR11-20, KR11-03, KR11-9014, 11KR-13, KR11-9025, KR12-07, GN-68/2003. Результаты этого исследо- вания представляют интерес для программы селекции пшеницы на устойчивость к пиренофорозу.

## Introduction

One of the main reasons for the reduction in yield of wheat in Kazakhstan are the diseases with airborne infection. Dominant position as a part of the pathogenic complex of wheat in the south and south-east of Kazakhstan took rusts (yellow, stem and leaf rust) (Kokhmetova et al., 2011, 2016b, 2018b; Rsaliyev A.S., Rsaliyev Sh.S., 2018), as well as leaf spot diseases (tan spot and Septoria) (Kokhmetova et al., 2017, 2018a, 2019).

The causative agent of wheat tan spot is the fungus Pyrenophora tritici-repentis, which belongs to the class Ascomycetes, the subclass of marsupials, the order Dothidiales, the family Pleorosporaceae. In addition to wheat, P. tritici-repentis infects more than 60 species of forage and wild-growing grasses (Koishybaev, 2010; Mironenko, Kovalenko, 2018). The infection is manifested on leaves and leaf sheaths of cereals in the form of small single or multiple spots oval or round shape, yellow or light-brown color, a chlorotic zone is formed around the spot. The source of the primary infection is the ascospores of the fungus, the secondary infection is caused by conidia, which are carried by the wind (Pospekhov, 1989).

The harmfulness of the disease leads to a decrease in the assimilation surface, an increase in transpiration, a decrease in the accumulation of organic matter, the defeat of all aboveground plant organs, a loss of grain quality due to the formation of unfulfilled grain. In conditions favorable for the development of the disease, losses of more than 50 % were noted (Shabeer, Bockus, 1988). P. tritici-repentis (PTR) (Died.), the causative agent of tan spot, induces two different symptoms on susceptible varieties – necrosis and chlorosis. Both symptoms are genetically under independent host control. At present, eight PTR races have been identified in the world based on the ability to induce symptoms of necrosis and chlorosis on a set of wheat differentiatial cultivars. Integrated disease control strategies, such as cultivation of resistant cultivars, combined with desired crop rotations and management practices, are the most effective, environmentally friendly and economical means to control wheat tan spot (Singh et al., 2010).

P. tritici-repentis is found in all major wheat-growing regions. The tan spot pathogen is registered in Australia, Canada, the United States of America, South America, Romania, Moldova, England, Kazakhstan, Ukraine, Belarus, Central Asia (Mikhailova et al., 2012). The first information about the distribution of P. tritici-repentis in Central Asia was presented by B.A. Khasanov in the early 1980s (Postnikova, Khasanov, 1997). Monitoring of wheat fields in Central Asia and Kazakhstan in 2003 showed that tan spot is most common on winter wheat, while the severity could reach from 50 to 100 % (Koishybaev, 2002; Lamari et al., 2005).

The compatibility reaction between the P. tritici-repentis race and the corresponding differential is realized through an intermediary – the host-specific toxin (Host Selective Toxins, HST). To date, four HSTs have been characterized: one toxin inducing necrosis, Ptr ToxA, two toxins inducing chlorosis, Ptr ToxB and Ptr ToxC, and one toxin inducing both necrosis and chlorosis, Ptr ToxD (Balance et al., 1989; Orolaza et al., 1995; Ali et al., 2010).

The population structure investigation of P. tritici-repentis in Kazakhstan have received attention since the beginning of the 2000s, and it continued in recent years (Zhanarbekova et al., 2005; Maraite et al., 2006; Kokhmetova et al., 2016b, 2017). The greatest diversity of racial composition in the pathogen population was noted in Azerbaijan, where races 1, 2, 3, 5, 7 and 8 were identified, and in Syria, where races 1, 3, 5, 7 and 8 were observed (Lamari et al., 2005 ). Race 1 was the most common in Central Asia and Kazakhstan (87 %), while races 2, 3, and 4 were less common (Zhanarbekova et al., 2005; Maraite et al., 2006). Earlier, we carried out a comparative study of the similarities and differences of P. tritici-repentis populations in terms of virulence and racial composition in the Republic of Kazakhstan and the North Caucasus region of Russia. It was shown that in recent years race 8 found in high frequency in Kazakhstan (Kokhmetova et al., 2016a, 2017).

The inheritance of resistance to tan spot is both quantitative and qualitative, and genes for resistance to toxins and quantitative trait loci (QTLs) are race-specific and control the process that reduces sensitivity to toxins (Mikhailova et al., 2012). Six major genes for resistance to tan spot Tsr1–Tsr6, localized on chromosomes 2BS, 3AS, 3BL, 3DS, and 5BL, have been identified (McIntosh et al., 2013). In the review by P.K. Singh et al. (2016) indicate that numerous genetic studies with the analysis of QTLs have demonstrated that resistance to tan spot is inherited as a polygenic trait, while the main racespecific genes, Tsr1 to Tsr6, often explain the effects of these loci (Singh S. et al., 2008; Singh P.K. et al., 2016). Additional QTLs have been identified and localized on chromosomes 1AL, 2AS, 3AS (Singh S. et al., 2008), 4AL, 5AL, 1BS, 2BL, 3BS, 3BL, 5BL, 2DS, 2DL and 7DS (Singh P. et al., 2016).

To increase the efficiency of breeding for resistance to tan spot, it is necessary to identify promising wheat lines characterized by a diversity of disease resistance genes, and then place them in the territory of the disease spread. Since under the influence of abiotic and biotic factors in nature there are permanent changes in the racial composition of pathogens, it is necessary to regularly analyze the structure of pathogen populations. This makes it possible to assess the dynamics of the variability of the racial composition in the population and to identify isolates with a new spectrum of virulence.

The aim of our research was to study the racial composition of the P. tritici-repentis population from the southeastern region of the Republic of Kazakhstan, as well as to search for sources of resistance to tan spot in the collection of wheat samples.

## Materials and methods

To determine the distribution area and harmfulness of Pyrenophora tritici-repentis, infected wheat leaf samples were randomly collected from winter bread wheat in the southeastern regions in 2018 in the Almaty region of the Republic of Kazakhstan. The analysis of the phytosanitary state of wheat crops was carried out during the period of heading and grain milk stages (June).

The object of the study was a collection of 30 entries of common wheat Triticum aestivum, including 17 promising breeding lines and cultivars from Kazakhstan and 13 entries from CIMMYT–ICARDA (see Table 2). The study of the wheat collection is aimed at finding the sources of resistance to PTR based on the assessment of seedling resistance to the dominant races of the fungus, the study of field (adult plant) resistance and molecular screening to P. tritici-repentis toxins. Salamouni (Lebanon) cultivar was used as a insensitive control for race 1 of tan spot and Ptr ToxA, Glenlea (Canada) – as a susceptible control for race 1 and Ptr ToxA.

Evaluation of field resistance to tan spot was carried out under conditions of the Kazakh Research Institute of Agriculture and Crop Production (KazNIIZiR), (Almalybak, 43°13′09ʺ N, 76°36′17ʺ E, Almaty region) in the 2019–2020 crop season. Experiments were conducted as a completely randomized design with two replicates in 1 m2. The severity of plants was assessed under conditions of an artificial infectious background on flag leaves in GS 65-69, Zadoks scale (Zadoks et al., 1974). The infectious background was created using infected with tan spot straw stubbles (1 kg/m2). The level of resistance was assessed according to the scale of 1–100 % for appraising the intensity of disease (Saari, Prescott, 1975). The standard wheat differentials of disease Glenlea (sensitive control) and Salamouni (resistant control) were used as controls.

The differentiation of races was carried out in accordance with the classification proposed by L. Lamari and C.C. Bernier (1998), using a Canadian set of disease differentials (wheat cultivar Glenlea and lines 6B662 and 6B365). The Ptr ToxA toxin induces the formation of necrosis symptoms the Glenlea wheat cultivar, and the Ptr ToxB and Ptr ToxC toxins induce the chlorosis symptoms on the 6B365 and 6B662 lines.

Monoconidial isolates of the fungus were isolated from wheat infectious material collected in the farm and breeding fields of the southeastern region of the Republic of Kazakhstan using the method of L.A. Mikhailova with colleagues (2012). To study the racial composition of the Kazakhstani population of P. tritici-repentis, 20 monoconidial isolates were used. The study of the structure of the population by racial composition and virulence was carried out using the method of leaf sections placed in a 0.004 % solution of benzimidazole (Mikhailova et al., 2012). Seedling resistance was also assessed using the benzimidazole method. The degree of development of the disease was assessed on the 7–8th day. The plants were rated for disease based on lesion type; cultivars with a necrotic reaction 1–2 were attributed to resistant (R), and with reaction 3–5 – to susceptible (S) entries (Lamari, Bernier, 1989). The presence or absence of chlorosis was assessed on lines 6B365 and 6B662.

Genomic DNA was extracted from 5-day-old wheat seedlings from plant material using the CTAB method (Riede, Anderson, 1996). To identify the carriers of resistance genes, the method of polymerase chain reaction (PCR) was used with primers flanking diagnostic gene markers and DNA samples from a collection of 30 common wheat samples (T. aestivum L.). The cultivars carrying the Tsn1 gene, which is sensitive to the Ptr ToxA toxin, were identified on the basis of PCR using the SSR marker Xfcp623 (Zhang et al., 2009; Faris et al., 2010). The marker has two alleles: 380 bp (associated with sensitivity, the dominant allele of the Tsn1 gene) and the null allele (associated with insensitivity, the recessive allele of the tsn1 gene) (Zhang et al., 2009). The composition of the reaction mixture and the PCR conditions followed the protocol (Roder et al., 1998). To separate the amplified DNA fragments, electrophoresis was performed in a 2 % agarose gel in TBE buffer (45 mM Tris borate, 1 mM EDTA, pH 8) (Chen et al., 1998). The gels were visualized using a Mega Bio-Print 1100/26M gel documenting system, Vilber Lourmat.

## Results

To study the racial composition of the Kazakhstani P. triticirepentis population, 20 monoconidial isolates were analyzed. Using differential lines and cultivars from Canada, 20 isolates were characterized as belonging to certain races of P. triticirepentis, presented in Table 1. In accordance with the generally accepted classification of races (Lamari et al., 1998), the isolates were assigned to race 1 as inducing toxins Ptr ToxA and Ptr ToxC, to race 2 (Ptr ToxA), race 3 (Ptr ToxC), race 4 (non-inducing toxins), race 5 (Ptr ToxB), race 6 (Ptr ToxB and Ptr ToxC), race 7 (Ptr ToxA and Ptr ToxB) and race 8 (Ptr ToxA, Ptr ToxB and Ptr ToxC).

**Table 1.**
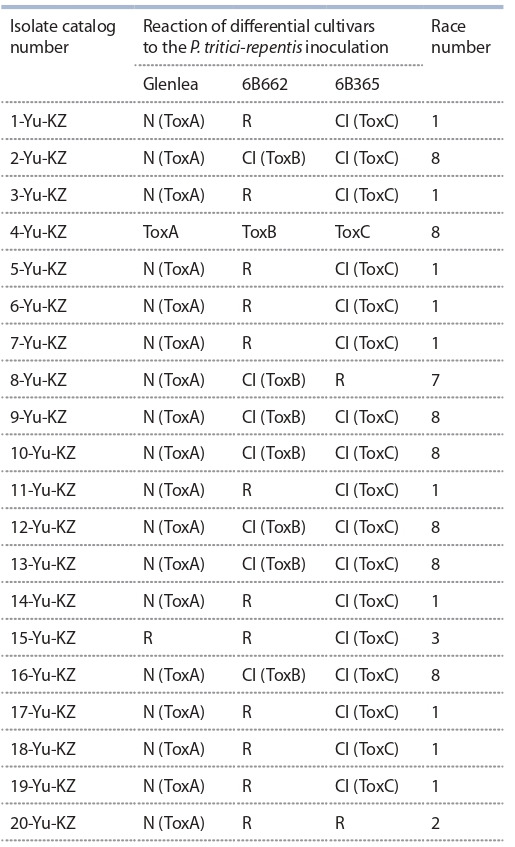
Determination of the races of P. tritici-repentis isolates from the south-east of Kazakhstan on the Canadian set of differential cultivars Notе. Manifestation of symptoms: N – necrosis, Cl – chlorosis; R – resistant response to P. tritici-repentis infection.

As a result of the analysis of the frequency of occurrence of the races of the pathogen P. tritici-repentis, it was found that in isolates from the southeast of Kazakhstan the dominant race was 1 producing Ptr ToxA and Ptr ToxB (50 %), and race 8 producing Ptr ToxA, Ptr ToxB and Ptr ToxC (35 %). Races 4, 5, and 6 were not found in the studied samples of the P. tritici-repentis population. Thus, in the southeastern region of Kazakhstan, five races of P. tritici-repentis have been identified: 1, 2, 3, 7, and 8.

Inoculation and evaluation of 30 promising lines and varieties of winter soft wheat in laboratory conditions were carried

**Table 2.**
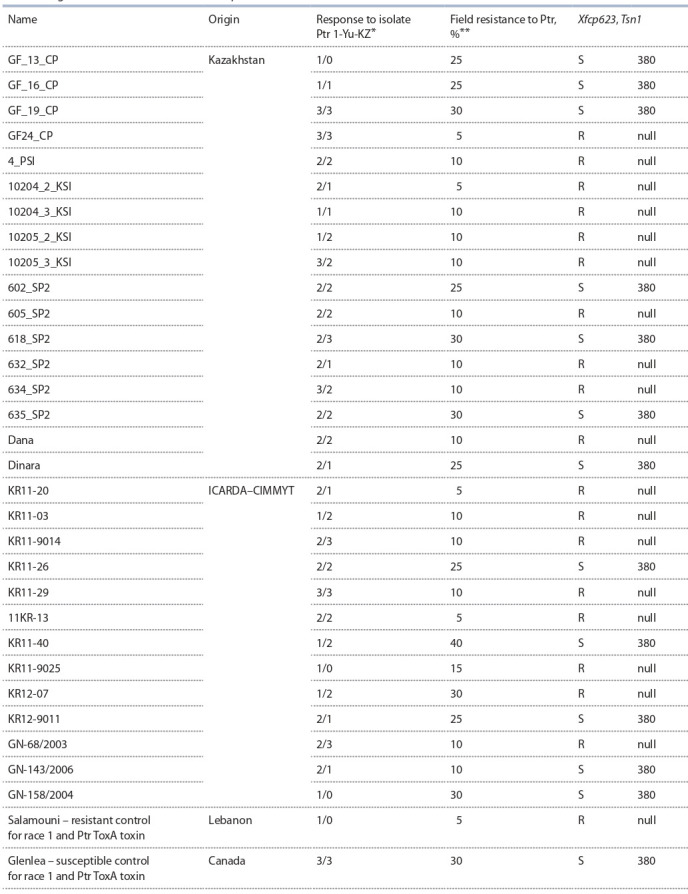
Screening of promising lines of winter bread wheat from Kazakhstan and ICARDA–CIMMYT for seedling and field resistance to P. tritici-repentis ^*^ Isolate 1-Yu-KZ, producing Ptr ToxA toxin; above the line – the score for necrosis; below the line – the score for chlorosis; ^**^ the average percent disease severity for two years are presented (2019–2020); Xfcp623 – SSR marker of the Tsn1 locus sensitive to Ptr ToxA amplifies a 380 bp DNA fragment.

The reactions of wheat genotypes to the Ptr 1-Yu-KZ isolate presented in Table 2 indicate that five promising lines are characterized by the highest resistance, which is 16.6 %. This group includes three Kazakhstani (GF_13_CP, GF_16_CP, 10204_3_KSI) and two foreign wheat lines from ICARDA–CIMMYT (KR11-9025 and GN-158/2004). Sixteen samples (53.3 %) showed moderate-resistant (MR) response. Moderately susceptible type of reaction (MS) was identified in three entries. The susceptible type of reaction (S) was observed in six entries, which is 20 % of the total amount of the studied material.

The results of assessing the field resistance to tan spot showed that the development of the disease was observed mainly in the lower and middle tiers of plants, the maximum severity was 40 %. A resistant type of reaction to the disease (10–15 %) was observed in 14 lines, which amounted to 46.6 % of the total amount of the studied material. Four wheat lines were characterized by high field resistance to the disease: GF24_CP, 10204_2_KSI, KR11-20 and 11KR-13.

The search for genotypes-carriers of alleles of the genes of sensitivity, Tsn1, and insensitivity, tsn1, to the Ptr ToxA P. tritici-repentis toxin in promising wheat lines was carried out as a result of molecular analysis of a collection of wheat entries and comparison of these results with screening based on reactions to isolate 1-Yu-KZ, producing Ptr ToxA toxin.

Genotyping of wheat entries using a molecular marker was aimed at identifying carriers of genes that control sensitivity and resistance to the Ptr ToxA toxin. The Xfcp623 marker amplified a 380 bp fragment associated with the Tsn1 gene sensitive to the Ptr ToxA toxin in 13 entries (43.3 %) (see Table 2, the Figure). The null allele of the Xfcp623 marker was found to be linked to toxin insensitivity in 17 wheat entries. Most of the entries (76.4 %) were insensitive to the isolate of race 1 (1-Yu-KZ) producing the Ptr ToxA toxin (see Table 2). The linkage rate of the Xfcp623 marker to race 1 insensitivity was 76.4 %. An example of an electrophoretogram with the results of PCR, reflecting the presence/absence of the Tsn1 locus sensitive to Ptr ToxA in the samples under study, is shown in the Figure.

**Fig. 1.**
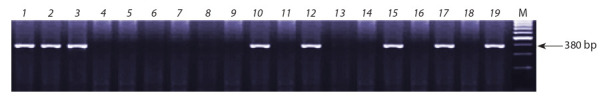
DNA amplification products of wheat entries using the diagnostic marker Xfcp623, linked to Tsn1 gene determining susceptibility to Ptr ToxA. 1 – GF_13_CP; 2 – GF_16_CP; 3 – GF_19_CP; 4 – GF24_CP; 5 – 4_PSI; 6 – 10204_2_KSI; 7 – 10204_3_KSI; 8 – 10205_2_KSI; 9 – 10205_3_KSI; 10 – 602_SP2; 11 – 605_SP2; 12 – 618_SP2; 13 – 632_SP2; 14 – 634_SP2; 15 – 635_SP2; 16 – Dana; 17 – Dinara; 18 – Saloumoni – resistant check to Ptr ToxA; 19 – Glenlea – susceptible check to PTR ToxA. M – 100 bp DNA Ladder (Gene-RulerTM, Fermentas). 2 % agarose gel.

The frequency of the Xfcp623 marker allele linked to the tsn1 gene, which controls the insensitivity to the Ptr ToxA toxin, was 56.6 % (17 entries). The frequency of the marker linked to the dominant allele of the Tsn1 gene linked to the sensitivity to the Ptr ToxA toxin was 43.3 % (13 entries).

The SSR marker Xfcp623 amplified a 380 bp DNA fragment associated with the dominant ToxA-sensitive Tsn1 allele in 13 entries and in the control Glenlea. Another allele, found using the Xfcp623 marker in other wheat entries (17), was a null allele characteristic of ToxA-insensitive genotypes and indicating the recessive state of the tsn1 allele.

From a practical point of view, of greatest interest are 16 wheat samples, which demonstrated resistance to race 1 and confirmed insensitivity to Ptr ToxA in a molecular screening. These include eight Kazakhstani (4_PSI, 10204_2_KSI, 10204_3_KSI, 10205_2_KSI, 10205_3_KSI, 605_SP2, 632_SP2, Dana) and seven foreign lines (KR11-20, KR11-03, KR11-9014, 11KR-13, KR11-9025, KR12-07, GN-68/2003).

## Discussion

The studies presented in this article made it possible to determine the racial composition of P. tritici-repentis isolates established in 2018 in the southeast of Kazakhstan. The study of the racial composition of the Kazakhstani southeastern population of P. tritici-repentis confirmed the previously obtained results (Kokhmetova et al., 2016a) and revealed the dominance of races 1 and 8 in this territory. An analysis of the frequency of occurrence of the fungus races showed that in the collection of isolates from Kazakhstan in 2018 the races 1 and 8 were dominant. In northern Kazakhstan in the early 2000s, races 1 (Zhanarbekova et al., 2005) and races 2, 3, and 4 (Maraite et al., 2006) were widespread. The indicated differences in the racial composition in Kazakhstan may be due to changes in climatic conditions in different years. The presented differences in the composition of the pathogen indicate the need for an annual analysis of the structure of pathogen populations in order to understand the dynamics of its variability and the distribution areas of P. tritici-repentis.

The study of the structure of tan spot population in three different climatic zones of Russia made it possible to determine the racial composition of the P. tritici-repentis populations. It was established that races 1 and 2 were dominant. Race 8 was found in all regions. The population from Dagestan lacked races 5, 6, 7, while the population from Western Siberia lacked race 4 (Kremneva et al., 2007; Mikhailova et al., 2010, 2014).

A comparative analysis of Kazakh and Russian samples of pathogen populations, carried out in 2016, showed that isolates from Kazakhstan are the most virulent, but the isolates from the North Caucasus region of Russia are the most phenotypically diverse (Kokhmetova et al., 2016a). The authors revealed a diversity in the virulence of isolates: in Russia, 4 races of P. tritici-repentis (1, 2, 4, and 8), and in Kazakhstan – five races (1, 3, 4, 6, and 8) were identified. Study of the population structure of P. tritici-repentis in the West Asian regions of Russia and North Kazakhstan showed that a high degree of similarity in the structure of fungi populations from these regions was noted on the basis of toxin formation, which indicates a single epidemiological zone (Gultyaeva et al., 2018).

Studies aimed at assessing the resistance of wheat germplasm to tan spot have received great attention (Chu et al., 2008; Singh P. et al., 2016; Kokhmetova et al., 2017, 2018a, 2019). The present work is due to the need to create genetically heterogeneous sources of resistance, which can be used in breeding wheat varieties resistant to P. tritici-repentis. This problem was solved on the basis of the use of DNA technologies and the use of isolates of race 1, which produce the most common toxin in Kazakhstan, Ptr ToxA.

Molecular markers for the diagnosis of insensitivity to Ptr ToxA and Sn ToxA (Xfcp393 and Xfcp394) have previously been developed (Zhang et al., 2009). After the Tsn1 gene was cloned and sequenced, the dominant SSR marker Xfcp623 was developed on the inner region of the gene (Faris et al., 2010). The Xfcp623 marker, proposed as a diagnostic marker for the Tsn1 gene, is considered more reliable than those previously developed. The reliability of the Xfcp623 diagnostic marker for detecting wheat genotypes with resistance to the pathogen and insensitivity to Ptr ToxA has been shown in a number of studies (Karelov et al., 2015; Mironenko et al., 2017). Taking into account the higher efficiency of the Xfcp623 marker, in our study we genotyped wheat germplasm using this marker.

As a result of our research, a collection of 30 common wheat entries was characterized using the Xfcp623 molecular marker, which is diagnostic for the Tsn1 gene associated with sensitivity to Ptr ToxA. From a practical point of view, of the greatest interest are 16 wheat samples, which demonstrated resistance to race 1 and confirmed resistance to Ptr ToxA in molecular screening. These include eight Kazakh and seven foreign wheat lines. Susceptibility to Ptr ToxA did not always correlate with susceptibility to race 1 and depended on the genetic background of the host.

The results of genotyping and screening of wheat entries for resistance to the most common isolates of P. tritici-repentis in Kazakhstan are of interest in order to increase the efficiency of breeding based on the elimination of carriers of dominant alleles of the Tsn1 gene sensitive to the aggressive Ptr ToxA toxin from the breeding material. Carriers of the identified tsn1 gene for resistance to Ptr ToxA can be used in breeding programs for gene pyramiding of genes for resistance to wheat diseases.

## Conflict of interest

The authors declare no conflict of interest.
